# The Role of Religion in Intergenerational Relations: Findings From the LSOG and WFDS Multigenerational Studies

**DOI:** 10.1093/geroni/igaf122.951

**Published:** 2025-12-31

**Authors:** J Jill Suitor, Megan Gilligan, Justin Hendricks

## Abstract

This symposium brings together scholarship that explores how religion shapes intergenerational relations in later-life families. The literature has documented that religion plays important roles in older adults’ health and well-being. However, little attention has been given to how religion shapes another major predictor of older adults’ well-being—the quality of their relationships with adult children and grandchildren. Papers in this symposium use data from two multi-wave panel studies of intergenerational relations (the Longitudinal Study of Generations and the Within-Family Differences Study) to examine how religion affects relationships between members of different generations. First, Silverstein and colleagues use LSOG data from 2016 and 2022 to examine the association between intergenerational religious discordance (e.g., regarding denomination, intensity, participation, belief in God) and closeness, contact, and support. Next, Ogle and colleagues use WFDS-III data to compare the association between adult children’s perceptions of religious value similarity to their mothers and relationship quality in later-life and mid-life families. Third, He and colleagues use data from the WFDS to explore stability in adult children’s perceptions of religious value similarity with their mothers and the role of similarity in the quality of parent-child relationships between 2008-2021. Last, Lakomy and colleagues use seven waves of the LSOG to study whether strength of religious values shapes grandparents’ provision of instrumental and emotional support to grandchildren, and the roles that lesser importance attributed to self-interest and individualistic goals play in mediating these associations. Hendrix will discuss these papers in the context of religion and family across the life course.

